# Interactions during falls with environmental objects: evidence from real-life falls in long-term care captured on video

**DOI:** 10.1186/s12877-024-05306-5

**Published:** 2024-09-02

**Authors:** Nataliya Shishov, Vicki Komisar, Daniel S. Marigold, Jean-Sébastien Blouin, Stephen N. Robinovitch

**Affiliations:** 1 Department of Biomedical Physiology and Kinesiology, Simon Fraser University, Burnaby, BC Canada; 2 Institute for Neuroscience and Neurotechnology, Simon Fraser University, Burnaby, BC Canada; 3 School of Kinesiology, University of British Columbia, Vancouver, BC Canada; 4 Djavad Mowafaghian Centre for Brain Health, University of British Columbia, Vancouver, BC Canada; 5 Institute for Computing, Information and Cognitive Systems, University of British Columbia, Vancouver, BC Canada

**Keywords:** Falls, Postural balance, Older adults, Long-term care, Environment, Video analysis

## Abstract

**Background:**

Falls are the leading cause of injuries in older adults. Environmental objects (such as furniture, walls, and handrails) may act as hazards or facilitators to balance maintenance and safe landing. There is lack of objective evidence on how older adults interact with objects during falls. We addressed this gap by characterizing body part contacts with objects other than the floor during real-life falls in long-term care.

**Methods:**

We analyzed videos of 1759 falls experienced by 584 residents to characterize the prevalence of contacts with objects before, during, and after fall initiation. Using generalized estimating equations, we compared the prevalence of falls with versus without contact to objects after fall initiation. Using linear mixed models, we tested for differences across body parts in the probability of contacting objects after fall initiation.

**Results:**

In nearly one-third of falls, interactions with objects (e.g., trips over objects, loss of support with objects) or with other people (e.g., being pushed by another person) had a primary role in causing imbalance and initiating the fall. After fall initiation, participants contacted objects in 60% of falls, with intentional hand contacts to objects via reach-to-grasp or bracing being the most common type of interaction (Probability ± SE = 0.32 ± 0.01), followed by unintentional impacts to the torso (0.21 ± 0.01) and head (0.16 ± 0.01). Intentional hand contact to an object was more common during forward than backward falls (*p < *0.001), while head and torso contacts to objects were more common during backward and sideways falls than forward falls (multiple *p* values ≤ 0.003). The hand most often contacted chairs, wheelchairs or couches, followed by tables or counters, walls, other people, walkers, and handrails. The head, torso, and shoulder most often contacted a wall.

**Conclusions:**

Most falls in long-term care involved contacts with objects other than the ground, indicating that complex environments often accompany falls in long-term care. Higher probabilities of intentional hand contacts in forward falls, versus unintentional head and torso impacts in backward and sideways falls may reflect the influence of being able to visualize and adjust one’s falling patterns to nearby objects.

**Supplementary Information:**

The online version contains supplementary material available at 10.1186/s12877-024-05306-5.

## Background

Falls are the number one cause of injuries in adults over age 65 [1]. The rates of falls and fall-related injuries are at least twice as common among residents of long-term care (LTC) than in older adults living independently [2–4]. An important component of falls management in LTC is the development of “safe movement environments” that reduce the risk for falls and fall-related injuries. To inform these efforts, we must better understand how environmental features (i.e., objects such as handrails, furniture, or walls) act as hazards or facilitators to balance maintenance, balance recovery, and safe landing [5, 6].

A barrier to the design of safe movement environments is the lack of objective evidence on how older adults interact with environmental objects during falls. Our knowledge of environmental interactions in real-life falls is limited to studies summarizing self-reports by the faller or witness (if any) on environmental contributors to imbalance [5–9]. These studies suggest that environmental factors have a primary role in causing most falls, through factors such as missing or inappropriate handrails, unstable or poorly positioned furniture, tripping or slipping hazards, and obstructed walkways. However, self-reported fall circumstances may be inaccurate and subject to recall bias [10]. Furthermore, few studies have considered environmental interactions beyond fall initiation, including reach-to-grasp attempts during descent [11], and body-part impacts to walls, furniture, or other objects. There is a need for a better understanding on how interactions with objects influence injury risk during falls, and how falls could be made safer through environmental design [9, 12, 13].

In the current study, we analyzed videos, collected from a 13 year observational study, of real-life falls experienced by older adults in common areas (dining rooms, hallways, and lounges) at two LTC facilities. We classified falls based on body part contacts to objects other than the ground before, during, and after fall initiation, and compared the probability of body parts in contacting objects. We also classified whether the contacts appeared to be intentional interactions (i.e., held objects, reach-to-grasp objects, or hand bracing on objects to arrest the fall) versus unintentional impacts between body parts and objects. We hypothesized that most falls in common areas of LTC result in contact of the body to objects other than the ground (Hypothesis 1), given that previous studies of self-reported fall circumstances found that environmental factors had a primary role in causing falls [5–9]. We also hypothesized that the prevalence of contacts to objects would differ across body parts and would depend on fall direction (Hypothesis 2). In particular, we expected that hand-to-object contacts would be more likely in forward falls, and head-to-object contacts would be more likely in backward falls. This hypothesis is based on two lines of evidence. First, body parts differ in their frequency of contact during falls, in a manner that depends on fall direction [14]. Second, vision allows one to tailor one’s falling patterns to environmental objects [15], and it seems reasonable to expect that people would be more able in forward than backward falls to visualize and coordinate hand contact to objects in the path of the fall, and avoid head contact to objects.

## Methods

### Participants and setting

This observational study was conducted between January 2007 and March 2020 at two LTC facilities in British Columbia, Canada [13, 14, 16]. Upon admission, all residents provided permission (by themselves or via a proxy decision maker) for the facility to obtain video footage in common areas (e.g., lounges, dining halls, and corridors) for safety purposes. Video footage of falls and corresponding fall incident reports were shared with our research team as secondary data. Most videos were recorded with a resolution of at least 640 × 480 pixels and a frame rate of 15–30 Hz. There were no cameras in bedrooms or bathrooms. A subset of participants (or their proxy decision makers) provided additional written informed consent to access their health records. This study was conducted in accordance with the Declaration of Helsinki. The Office of Research Ethics at Simon Fraser University (protocol number H21-00741) and the equivalent review board in the Fraser Health Authority reviewed and approved the study protocol.

### Fall inclusion criteria

**Fig. 1:**
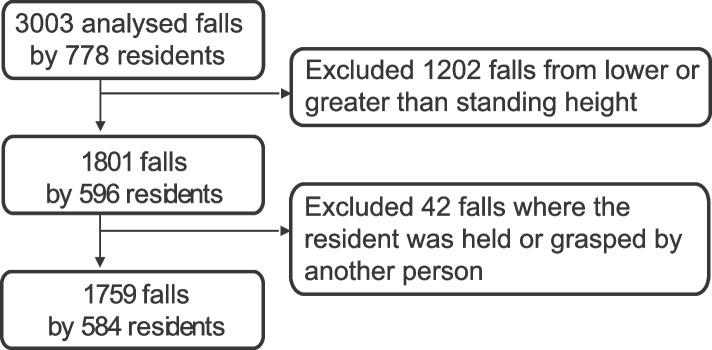
Flow chart of video selection process

Over the period of the study, we collected 3003 falls by 778 residents over the age of 65 (who we hereafter refer to as “participants”; Fig. 1). A team of three trained raters analyzed each video using a structured questionnaire to classify characteristics of the initiation, descent, and impact stages of the fall [17]. For each question, the team agreed on the best available response. In the current study, we only analyzed falls from standing height, which present a different context, and are more likely to result in injury when compared to falls from a sitting or lying position [18–20]. Accordingly, we excluded falls from lower than standing height (*n* = 1199), and falls from greater than standing height (*n = * 3). For cases where there were interactions between the faller and another person during the fall, we included cases where the faller held, reached-to-grasp, or otherwise initiated contact with the other person. However, we excluded cases (*n =* 42) where participants were held or grabbed by another person during the fall, since these contacts were not initiated or controlled by the faller. The resulting dataset used in our analyses included 1759 falls by 584 participants.

### Participant-object interactions during falls

Based on our video analysis, we classified four types of interactions between participants and objects during falls: (a) interactions with objects that were perceived to have a primary role in causing imbalance leading to the fall; (b) held objects at the time of fall initiation; (c) hand contacts to objects after fall initiation that appeared to be intentional, including reach-to-grasp movements or bracing of the hands on objects to arrest the fall; and (d) impact after fall initiation between objects and any part of the body (e.g. head, torso, shoulder, pelvis/hip, knee, elbow/forearm, and hand/wrist), that were not due to reach-to-grasp movements or hand bracing, and generally appeared to be unintentional. Only hand contacts with objects had the potential to be classified as intentional, whereas all other body part contacts were considered unintentional.

We defined “objects” as fixed features of the environment, or movable entities that rested on a fixed feature of the environment. We classified objects as: (1) chair, couch, or wheelchair; (2) wall or face of a door or of a counter, (3) table or edge of counter, (4) walker or rollator, (5) handrail, (6) another person, and (7) other. We classified the impacting body part as head, pelvis/hip, torso, hand/wrist, elbow/forearm, shoulder, or knee. We classified the initial fall direction as forward, sideways, or backward. In cases where the initial fall direction was classified as straight-down, the fall direction was classified (as forward, sideways, or backward) based on the body configuration at landing.

### Reliability of video analyses

In developing the questionnaire, we assessed the inter-rater reliability of each question by comparing responses (based on kappa values) from two independent teams of raters (with 3 raters per team) who analyzed the same 15 videos. We also assessed intra-rater reliability by comparing responses from the same team of raters in analyzing 15 videos at two time points separated by a year [17]. A minimal required sample of 15 videos was calculated based on guidelines for observer agreement studies [21]. We estimated that the average agreement between teams (for a given question) would be 85 percent, or 15 percent disagreement. We calculated that a sample size of 15 falls would allow us to detect a desired 90% confidence interval of 0 to 30 percent disagreement between teams [17]. Questions regarding body part impacts had good inter-rater reliability for the head, torso, and shoulder (Cohen’s Kappa ≥ 0.60, percent agreement ≥ 80%), and outstanding reliability for the pelvis/hip, hand/wrist, elbow/forearm, and knee (≥ 0.84, ≥ 93%). Intra-rater reliability was moderate for impact to the torso (0.41, 67%), good for the hand/wrist (0.67, 87%), and outstanding for the head, pelvis/hip, elbow/forearm, knee, and shoulder (≥ 0.82, ≥ 93%). The occurrence of reach-to-grasp had moderate inter-rater (0.44, 80%) and intra-rater (0.44, 87%) reliability. Held objects had moderate inter-rater reliability (0.33, 73%) and outstanding intra-rater reliability (1.0, 100%).

### Health status

A subset of 193 participants (accounting for 643 falls, 36.6%) provided consent to access their medical records. Information on disease diagnoses, medications, and visual status was collected from the Minimum Data Set (MDS 2.0; interRAI Corporation 1999), which was updated quarterly by nursing staff at the facilities [22]. MDS data were used to determine physical and cognitive status based on the Activities of Daily Living (ADL) Self-Performance Hierarchy Scale [23] and  the Cognitive Performance Scale (CPS) [24], respectively. For each fall, we collected health status information from the MDS assessment conducted prior and closest to the date of the fall, which we regarded as most representative of health status at the time of the fall. While the majority of health status variables were updated quarterly by nursing staff, visual impairment and disease diagnoses other than diabetes and stroke were updated annually. The average time interval between the MDS assessment and the date of the fall was 51.7 days (SD = 35.6, range = 0–267) for ADL and CPS assessments, diabetes and stroke diagnoses, and use of medications. The average time interval was 101.2 days (SD = 100.1, range = 0–533) for visual impairment, and disease diagnoses other than diabetes and stroke.

### Statistical analyses

We provide descriptive statistics on the frequency of human-environmental interactions during falls, including held objects and contact by body parts to objects after fall initiation, and the types of objects involved in these interactions. We addressed Hypothesis 1 (that most falls result in contacts with objects) using Generalized Estimating Equations (GEE; SPSS, version 25; IBM Corporation, NY) to test whether there were differences between environmental classifications in the odds that (a) a participant would fall at least once for a given classification (using binary logistic regression), and (b) the average number of falls per participant for a given classification (using log-linear Poisson regression). Environmental classifications included whether there was contact with an object after fall initiation; whether contact with an object after fall initiation, if present, was intentional or unintentional; and whether the person (also) grasped held objects. We report odds ratios (OR) and 95% confidence intervals (CI) for all GEE analyses. We addressed Hypothesis 2 (that the prevalence of contacts to objects would differ across body parts) using binary logistic regression linear mixed models (MIXED Procedure, SAS Version 9.4, Cary, NC) to test for differences in the probability of body parts contacting an object other than the floor after fall initiation. We regarded our sample size as sufficient for testing these hypotheses, based on our previous analysis using similar statistical approaches. For example, in a previous study that used a sample size nearly tenfold smaller than the current study (227 versus 1759 falls), we were able to detect differences smaller than 10% between categories in the proportion of participants falling, using a similar GEE-based approach [16]. In a more recent study that had a sample size similar to the current study (2388 falls), we were able to detect differences as small as 5% in the probability to contact to different body parts, using binary logistic regression linear mixed models [25].

As fall direction influences injury risk and protective responses for balance recovery and safe landing [25–27], we examined how the prevalence of contacts to objects in the different environmental classifications and by different body parts depended on fall direction, by running separate GEE models with only forward falls, sideways falls, or backward falls and by including fall direction as an exploratory variable in the linear mixed models. Furthermore, we included sex and age (below versus above median age) as covariates in GEE models and as exploratory variables in mixed models, given that each of these variables influence the rates of falls and fall-related injuries [14, 28–30]. We included Participant ID as a random factor in all analyses to account for repeated falls in the same participants, and we used the “exchangeable” correlation matrix structure in all GEE analyses. We used a level of significance of *p < *0.05 for all analyses.

## Results

### Participant characteristics

For the 584 participants included in our analysis, 55% were females and the mean age at time of first fall captured on video was 83.8 years (SD = 8.0). Among the 1759 falls included in the analyses, 1004 (57%) were experienced by females and 755 by males. The 193 participants who provided consent to assess their medical records experienced a total of 643 falls (Table 1). Of these falls, 79% were experienced by participants who had moderate to very severe cognitive impairment, 67% by participants dependent in their ADLs, and 28% by participants with impaired vision. The most common disease diagnosis was hypertension (48% of falls), followed by Alzheimer’s disease (34%) and diabetes (18%).

**Table 1:** Characteristics of participants who experienced 643 falls and provided consent for access to medical records

	All falls included in the analyses with MDS^a^ (*n =* 643)	Falls that did not involve contacted objects (*n =* 128)^h^	Falls that involved held objects (*n =* 324)^i^	Falls that involved intentional hand contact to objects (*n =* 201)^j^	Falls that involved unintentional impact to objects (*n =* 290)^k^
**Demographics and health status**					
Age—older than median n (%)^b^	309 (48.1%)	38 (29.7%)	180 (55.6%)*	99 (49.3%)*	151 (52.1%)*
Sex—female, n (%)^c^	429 (66.7%)	84 (65.6%)	211 (65.1%)	130 (64.7%)	188 (64.8%)
Dependent ADL^d^ performance, n (%)	433 (67.3%)	85 (66.4%)	213 (65.7%)	139 (69.2%)	202 (69.7%)
Moderate to severe cognitive impairment^e^, n (%)	510 (79.3%)	105 (82.0%)	246 (75.9%)	164 (81.6%)	238 (82.1%)
Impaired vision^f^, n (%)	180 (28.0%)	37 (28.9%)	90 (27.8%)	57 (28.4%)	82 (28.3%)
**Disease diagnoses**					
Diabetes, n (%)	114 (17.7%)	15 (11.7%)	58 (17.9%)	45 (22.4%)*	58 (20.0%)
Cardiac dysrhythmia, n (%)	44 (6.8%)	14 (10.9%)	15 (4.6%)	17 (8.5%)	23 (7.9%)
Congestive heart failure, n (%)	21 (3.3%)	3 (2.3%)	11 (3.4%)	7 (3.5%)	8 (2.8%)
Hypertension, n (%)	309 (48.1%)	49 (38.3%)	171 (52.8%)	100 (49.8%)	144 (49.7%)
Hypotension, n (%)	30 (4.7%)	10 (7.8%)	13 (4.0%)	13 (6.5%)	13 (4.5%)
Alzheimer’s disease, n (%)	219 (34.1%)	57 (44.5%)	77 (23.8%)	72 (35.8%)	100 (34.5%)
Stroke, n (%)	69 (10.7%)	7 (5.5%)	41 (12.7%)*	24 (11.9%)	38 (13.1%)*
Parkinson’s disease, n (%)	44 (6.8%)	10 (7.8%)	29 (9.0%)	16 (8.0%)	13 (4.5%)
Emphysema /COPD^g^, n (%)	74 (11.5%)	9 (7.0%)	46 (14.2%)	19 (9.5%)	29 (10.0%)
Cataract, n (%)	85 (13.2%)	17 (13.3%)	52 (16.0%)	22 (10.9%)	32 (11.0%)
Glaucoma, n (%)	54 (8.4%)	6 (4.7%)	32 (9.9%)	20 (10.0%)	28 (9.7%)
Macular degeneration, n (%)	35 (5.4%)	4 (3.1%)	20 (6.2%)	7 (3.5%)	20 (6.9%)
**Use of medications**					
Antipsychotics, n (%)	231 (35.9%)	55 (43.0%)	100 (30.9%)*	78 (38.8%)	95 (32.8%)*
Antianxiety agents, n (%)	107 (16.6%)	16 (12.5%)	51 (15.7%)	40 (19.9%)	54 (18.6%)
Antidepressants, n (%)	332 (51.6%)	72 (56.3%)	154 (47.5%)	114 (56.7%)	155 (53.4%)
Hypnotics, n (%)	125 (19.4%)	15 (11.7%)	70 (21.6%)	47 (23.4%)	67 (23.1%)
Diuretics, n (%)	105 (16.3%)	19 (14.8%)	57 (17.6%)	29 (14.4%)	44 (15.2%)
Analgesics, n (%)	245 (38.1%)	38 (29.7%)	136 (42.0%)	83 (41.3%)	106 (36.6%)

NOTES AND ABBREVIATIONS:

^a^MDS = Data in this table is based on the “Minimum Data Set “ assessment

^b^Age was available for all 584 participants included in the study. The mean (SD) participant age was 83.8 (8.0) years, and the median age was 85 years

^c^Sex was available for all 584 participants included in the study, 324 (55.5%) of whom were female

^d^ADL – “Activities of Daily Living”. Scores of 0–2 were classified as “independent” and scores of 3–6 were classified as “dependent”

^e^Cognitive impairment was assessed using the “Cognitive Performance Scale”. Scores of 0–2 were classified as “intact to mild cognitive impairment” and scores of 3–6 were classified as “moderate to very severe cognitive impairment”

^f^Visual impairment – Score of 0 in the MDS was classified as “no visual impairment” and scores of 1–4 were classified as “visual impairment”

^g^COPD – “Chronic Obstructive Pulmonary Disease”

^h^falls that did not involve any interactions or contacts to objects

^i^falls that involved held objects at the time of fall initiation

^j^falls that involved hand contacts to objects after fall initiation that appeared to be intentional, including reach-to-grasp movements or bracing of the hands on objects to arrest the fall

^k^falls that involved impact after fall initiation between objects and any part of the body (e.g. head, torso, shoulder, pelvis/hip, knee, elbow/forearm, and hand/wrist), that were not due to reach-to-grasp movements or hand bracing, and generally appeared to be unintentional

^h^falls that did not involve any interactions or contacts to objects

^*^Significant association with contact to object category versus no object (*p ≤ *.05), based on generalized estimating equation comparisons

The columns with different types of contacts to objects (right three) include all falls for each category. Accordingly, falls with more than one type of contact may appear in more than one of these columns

There were few differences in the demographics and the clinical characteristics of participants who fell and contacted objects, and those who fell and did not contact objects (Table 1). When compared to falls with no contact to objects, the proportion of falls that involved contact to objects was higher in participants older than the median age (56% versus 30%, *p =* 0.005 for held objects; 49% versus 30%, *p =* 0.004 for intentional hand contacts; and 52% versus 30%, *p < *0.001 for unintentional impacts). The proportion of falls that involved held objects and unintentional impact to objects was also higher in participants with stroke (13% versus 6%, *p ≤ *0.024), and lower in participants who used antipsychotics (31% and 33% versus 43% respectively, *p ≤ *0.028). Finally, the proportion of falls that involved intentional hand contact to objects was higher in participants with diabetes (22% versus 12%, *p =* 0.045).

### Interactions with objects causing a fall

In nearly one-third of falls, interactions with objects or other people before fall initiation had a primary role in causing imbalance leading to the fall. Trips caused 21% of falls (*n =* 374), and 51% of trips (*n =* 190) involved the foot becoming caught on an object, most often a chair/couch/wheelchair (*n =* 85), walker/rollator (*n =* 62), person (*n =* 16), floor surface transition (e.g., raised edge of a carpet) (*n =* 11), other (*n =* 11), or table/counter (*n =* 5). The 49% of trips that were not caused by interactions with objects involved the foot being caught on level ground (*n =* 119) or on the faller’s other foot (*n =* 65). Loss of support with an external object contributed to 7% of falls (*n =* 122). Of these, the most common scenario was loss of support with a chair/couch/wheelchair (*n =* 49), walker/rollator (*n =* 49), other (*n =* 9), another person (*n =* 7), table/counter (*n =* 6), wall (*n =* 1), and handrail (*n =* 1). In 13% of falls (*n =* 221), the faller was pushed or pulled by another person, and 2% of falls (*n =* 37) involved bumping into another person or object.

### Held objects at fall initiation

Participants held objects at fall initiation in 51% of falls (*n =* 898). Objects were held in both hands in 519 falls, and in only one hand in 379 falls. The most commonly held object was a walker/rollator (*n =* 339), followed by a chair/couch/wheelchair (*n =* 292), person (*n =* 132), table/counter (*n =* 123), other (*n =* 53), handrail (*n =* 38), and a wall (*n =* 13).

### Interactions with objects initiated after the onset of the fall

**Fig. 2:**
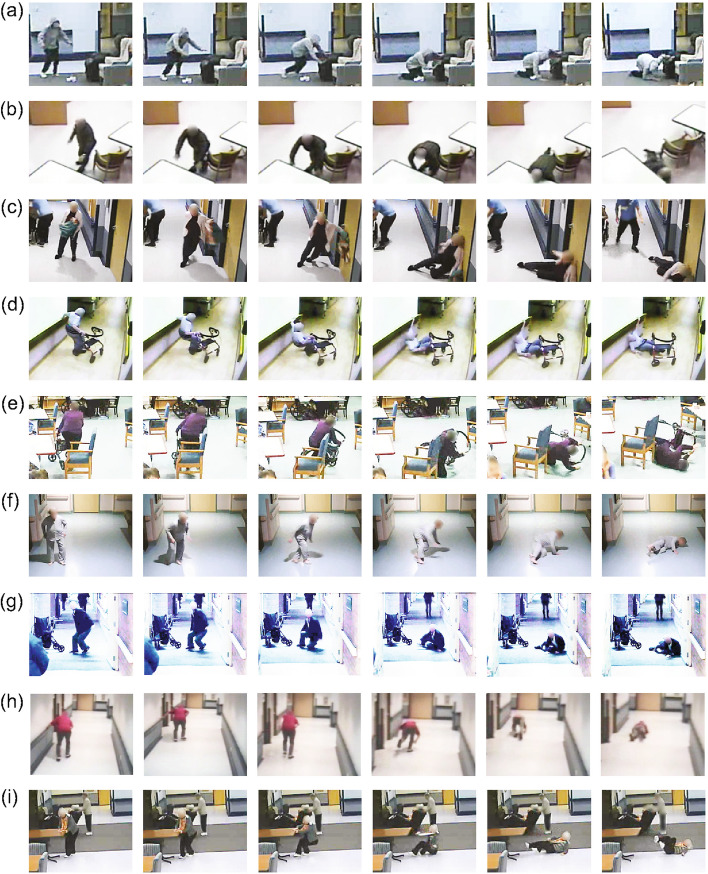
Snapshots from videos of real-life falls. Videos display different types of interactions between participants and environmental objects. **a**-**e** Falls with contacts after the onset of the fall to the (**a**, **b**) forward, (**c, e**) sideways, and (**d**) backward directions. Falls (**a**) and (**c**) involved intentional hand contacts with (**a**) a chair, and (**c**) a door, whereas falls (**b**-**e**) involved unintentional impacts of the (**c**, **d**) torso, (**d**) head, and (**e**) pelvis to (**c**) a door, (**d**) a wall, and (**e**) a chair. Falls (**d**) and (**e**) also involved held (**d**, **e**) walker and (**d**) a handrail. (**f**, **g**) Sideways falls without contact to objects. (**h**, **i**) Falls with only held (**h**) handrail and (**i**) table before the onset of the fall

**Fig. 3:**
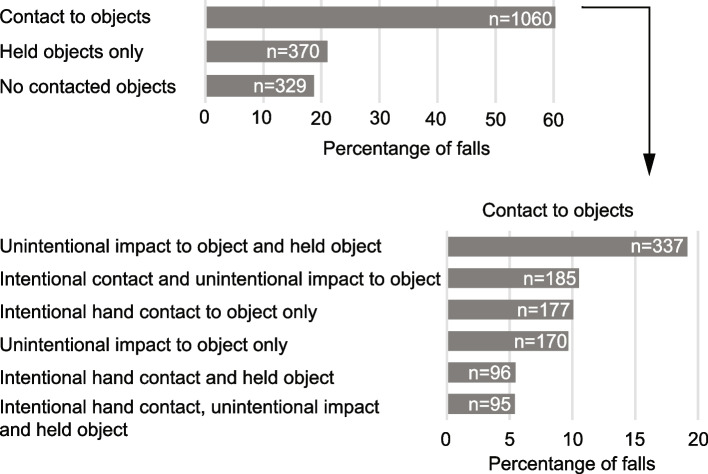
Distribution of falls with different types of interactions with objects

Contact with objects was initiated after the onset of the fall in 60% of falls (*n =* 1060; Figs. 2 and 3). 16% of falls (*n =* 273) involved only intentional hand contacts to objects, 29% (*n =* 507) of falls involved only unintentional contacts to objects, and 16% (*n =* 280) involved both intentional hand contact and unintentional impact by at least one body part. Among these three groups of falls, 96, 337 and 95 falls also involved held objects, respectively. Of the 699 falls not involving contact to objects after the onset of the fall, 370 (21% of all falls) involved held objects at fall initiation, whereas 329 (19% of falls) involved no interactions with objects at or after fall initiation.

### Odds for contacting objects after fall initiation

Falls were more likely to involve contact to objects initiated after the onset of the fall (Table 2). The estimated proportion of participants who fell and contacted an object was greater than the proportion falling without contacting an object (0.77 versus 0.60, *p < *0.001). The average number of falls per participant was also greater for falls where objects were (versus were not) contacted (1.80 versus 1.19; *p < *0.001). These trends held for all fall directions except for forward falls, where there were no differences in the proportion of participants who fell and contacted (versus did not contact) an object [see Additional files 1–3].

**Table 2:** Odds that a participant would contact objects during falls

Environment	Number of falls	Frequency (% of falls)	Proportion (of the 584 participants) who fell at least once	Odds ratio (95%CI)	*P* value	Average number of falls per participant	Ratio of counts (95%CI)	*P* value
**(a)**								
Contacted objects after fall initiation^j^^, k^	1060	60.3	0.77 (0.73–0.80)	2.14 (1.66–2.75)	***p < *0.001**	1.80 (1.62–2.01)	1.52 (1.37–1.68)	***p < *0.001**
Did not contact objects after fall initiation^h^	699	39.7	0.60 (0.56–0.64)	1	…	1.19 (1.05–1.35)	1	◊
**(b)**								
Did not contact objects after fall initiation^h^	699	39.7	0.60 (0.56–0.64)^C^	1.48 (1.17–1.86)	***p =* 0.001**	1.18 (1.04–1.34)^C^	1.38 (1.22–1.55)	***p < *0.001**
Intentionally contacted objects after fall initiation^j^	273	15.6	0.33 (0.29–0.37)^A^	0.48 (0.38–0.61)	***p < *0.001**	0.46 (0.39–0.54)^A^	0.54 (0.46–0.63)	***p < *0.001**
Intentionally^j^ AND unintentionally^k^ contacted objects after fall initiation	280	15.9	0.31 (0.27–0.35)^A^	0.43 (0.34–0.55)	***p < *0.001**	0.47 (0.40–0.56)^A^	0.55 (0.47–0.65)	***p < *0.001**
Unintentionally impacted objects after fall initiation^k^	507	28.8	0.51 (0.47–0.55)^B^	1	…	0.86 (0.76–0.96)^B^	1	◊
**(c)**								
Contacted objects after fall initiation^j^^, k^ AND held object^i^	528	30.0	0.53 (0.49–0.57)^C^	2.52 (1.99–3.20)	***p < *0.001**	0.90 (0.80–1.00)^B^	1.61 (1.32–1.95)	***p < *0.001**
Contacted objects after fall initiation^j^^, k^ AND did not hold object^g^	532	30.3	0.47 (0.43–0.51)^B^	1.94 (1.53–2.47)	***p < *0.001**	0.90 (0.78–1.05)^B^	1.62 (1.38–1.90)	***p < *0.001**
Did not contact objects after fall initiation^h^ AND held object^i^	370	21.0	0.42 (0.38–0.46)^B^	1.59 (1.25–2.02)	***p < *0.001**	0.63 (0.55–0.72)^A^	1.13 (0.90–1.41)	*p =* 0.302
Did not contact objects after fall initiation^h^ AND did not hold object^g^	329	18.7	0.31 (0.27–0.35)^A^	1	…	0.56 (0.46–0.68)^A^	1	◊

-Significant differences (*p < *0.05) in the odds that a participant would fall at least once between environments, and differences in the average number of falls between environments are bolded

-Superscript capital letters indicate the results of statistical comparisons between environmental classifications. Environmental classifications that differed significantly (*p < *0.05) are indicated by different letters; environmental classifications that did not differ (*p > *0.05) are indicated by the same letter. The sequence of the letters is from lowest to highest proportions and average number of falls. For example, the proportion of participants who fell at least once and “Did not contact objects after fall initiation AND did not hold object” (0.31 (0.27–0.35)^A^) was significantly smaller than all other categories. The proportion of participants who fell at least once and “Did not contact objects after fall initiation AND held object” (0.42 (0.38–0.46)^B^) was significantly larger than the proportion of participants who fell at least once and “Did not contact objects after fall initiation AND did not hold object” (0.31 (0.27–0.35)^A^), but was not different than the proportion of participants who fell at least once and “Contacted objects after fall initiation AND did not hold object” (0.47 (0.43–0.51)^B^). The proportion of participants who fell at least once and “Contacted objects after fall initiation AND held object” (0.53 (0.49–0.57)^C^) was significantly larger than all other categories

-Superscript lower case letters indicate the types of interactions with objects included in each category:

^g^falls that did not involve held objects at the time of fall initiation

^h^falls that did not involve any interactions or contacts to objects after fall initiation (may have involved held objects at the time of fall initiation)

^i^falls that involved held objects at the time of fall initiation

^j^falls that involved hand contacts to objects after fall initiation that appeared to be intentional, including reach-to-grasp movements or bracing of the hands on objects to arrest the fall (in comparisons (a) and (b), these falls may have also involved held objects at the time of fall initiation)

^k^falls that involved impact after fall initiation between objects and any part of the body (e.g. head, torso, shoulder, pelvis/hip, knee, elbow/forearm, and hand/wrist), that were not due to reach-to-grasp movements or hand bracing, and generally appeared to be unintentional (in comparisons (a) and (b), these falls may also have involved held objects at the time of fall initiation)

◊Significant effect of age as a covariate (*p < *0.05)

Falls involving contact to an object after fall initiation were more likely to involve unintentional than intentional impact to objects (Table 2). The estimated proportion of participants who fell and unintentionally impacted an object was higher than the proportions who intentionally contacted an object, or fell and both intentionally and unintentionally contacted objects in the same fall (0.51 versus 0.33 and 0.31, *p < *0.001). The average number of falls per participant was also higher for falls where objects were contacted unintentionally versus intentionally (0.86 versus 0.46, *p < *0.001). These trends held for all fall directions except for forward falls, where there were no differences in the proportion of participants who fell and unintentionally versus intentionally contacted an object [see Additional files 1–3].

Of the participants who fell while holding an object, a higher proportion also contacted an object after fall initiation (0.53 versus 0.42, *p < *0.001). This trend held for backward and sideways falls, but not for forward falls [see Additional files 1–3].

### Differences in probability of body parts contacting objects

**Fig. 4:**
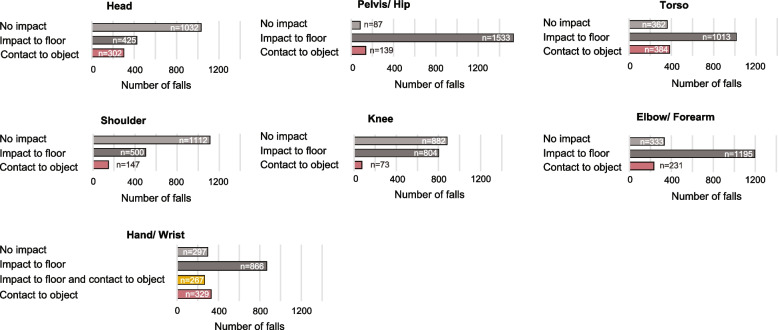
Distribution of impacts to body parts. The figure displays the prevalence of falls where specific body parts did not impact any surface, impacted only the floor, or contacted an object

The probability of distinct body parts contacting objects differed during falls (*p < *0.001; Table 3 and Fig. 4). The most common type of interaction was intentional contact of the hand/wrist with objects (Probability ± SE = 0.32 ± 0.01), followed by unintentional impacts of the torso (0.21 ± 0.01), head (0.16 ± 0.01), elbow/forearm (0.13 ± 0.01), shoulder (0.08 ± 0.01), pelvis/hip (0.08 ± 0.01), knee (0.04 ± 0.01) and hand/wrist (0.04 ± 0.01). Pairwise comparisons showed differences between all body parts (multiple *p* values ≤ 0.003), except for the pelvis and shoulder (*p =* 0.742), and for the knee and unintentional hand/wrist impacts (*p =* 0.748).

**Table 3:** Estimated probability of body part contact to objects other than the floor after fall initiation

Body part	All falls (SE) *n* = 1759	Forward falls (SE) *n* = 420	Backwards falls (SE) *n* = 678	Sideways falls (SE) *n* = 661	*p* value forward versus backward	*p* value forward versus sideways	*p* value backward versus sideways	Falls by younger half of participants (SE) *n* = 908	Falls by older half of participants (SE) *n* = 851	*p* value age	Falls by males (SE) *n* = 755	Falls by females (SE) *n* = 1004	*p* value sex
Head	0.16 (0.01)^C^ *n* = 302	0.12 (0.02)^B^ *n* = 49	0.19 (0.01)^B^ *n* = 134	0.18 (0.01)^B^ *n* = 119	**< .001**	**.003**	.374	0.14 (0.01)^C^ *n* = 134	0.18 (0.01)^C^ *n* = 168	**.012**	0.15 (0.01)^C^ *n* = 110	0.18 (0.01)^C^ *n* = 192	**.028**
Torso	0.21 (0.01)^B^ *n* = 384	0.12 (0.02)^B^ *n* = 49	0.28 (0.01)^A^ *n* = 194	0.21 (0.01)^B^ *n* = 141	**< .001**	** < .001**	**< .001**	0.18 (0.01)^B^ *n* = 170	0.23 (0.01)^B^ *n* = 214	**.001**	0.20 (0.01)^B^ *n* = 156	0.21 (0.01)^B^ *n* = 228	.641
Shoulder	0.08 (0.01)^E^ *n* = 147	0.05 (0.02)^CD^ *n* = 22	0.08 (0.01)^CD^ *n* = 55	0.11 (0.01)^CD^ *n* = 70	.160	**.010**	.173	0.08 (0.01)^D^ *n* = 73	0.08 (0.01)^E^ *n* = 74	.795	0.08 (0.01)^D^ *n* = 60	0.08 (0.01)^D^ *n* = 87	.816
Pelvis/Hip	0.08 (0.01)^E^ *n* = 139	0.05 (0.02)^CD^ *n* = 19	0.11 (0.01)^C^ *n* = 72	0.07 (0.01)^D^ *n* = 48	**.003**	.183	.055	0.08 (0.01)^D^ *n* = 71	0.08 (0.01)^E^ *n* = 68	.950	0.09 (0.01)^D^ *n* = 66	0.07 (0.01)^DE^ *n* = 73	.250
Knee	0.04 (0.01)^F^ *n* = 73	0.04 (0.02)^CD^ *n* = 18	0.06 (0.01)^D^ *n* = 37	0.03 (0.01)^E^ *n* = 18	.581	.422	.121	0.04 (0.01)^E^ *n* = 32	0.05 (0.01)^EF^ *n* = 41	.349	0.05 (0.01)^DE^ *n* = 37	0.03 (0.01)^E^ *n* = 36	.252
Elbow/Forearm	0.13 (0.01)^D^ *n* = 231	0.08 (0.02)^BC^ *n* = 34	0.17 (0.01)^B^ *n* = 115	0.12 (0.01)^C^ *n* = 82	**< .001**	.053	**.013**	0.10 (0.01)^D^ *n* = 96	0.15 (0.01)^D^ *n* = 135	**.004**	0.12 (0.01)^C^ *n* = 92	0.13 (0.01)^C^ *n* = 139	.729
Hand/Wrist Intentional	0.32 (0.01)^A^ *n* = 553	0.36 (0.02)^A^ *n* = 150	0.28 (0.01)^A^ *n* = 189	0.32 (0.01)^A^ *n* = 214	**< .001**	.104	**.014**	0.32 (0.01)^A^ *n* = 286	0.32 (0.01)^A^ *n* = 267	.738	0.33 (0.01)^A^ *n* = 241	0.32 (0.01)^A^ *n* = 312	.509
Hand/Wrist Unintentional	0.04 (0.01)^F^ *n* = 69	0.04 (0.02)^D^ *n* = 16	0.04 (0.01)^D^ *n* = 27	0.04 (0.01)^E^ *n* = 26	.960	.986	.971	0.03 (0.01)^E^ *n* = 29	0.05 (0.01)^F^ *n* = 40	.335	0.04 (0.01)^E^ *n* = 29	0.04 (0.01)^E^ *n* = 40	.819

-Table includes least square mean estimates, standard errors (SE), pairwise comparisons for differences between body parts, and effect of fall direction, age, and sex on probability of body part contacts

- Superscript letters indicate the results of statistical comparisons between body parts for contact to objects other than the floor. Body parts that differed significantly (*p < *0.05) are indicated by different letters; body parts that did not differ (*p > *0.05) are indicated by the same letter. The sequence of letters is from highest to lowest in the estimated probability of contact. For example, when considering all falls, intentional contact of the hand/wrist had the highest probability (0.32(0.01)^A^), followed by contact of the torso (0.21(0.01)^B^). When considering only backward falls, probabilities of intentional contact of the hand/wrist (0.28 (0.01)^A^) and contact of the torso (0.28 (0.01)^A^) did not differ significantly, but were significantly higher than the probability of head contact (0.19 (0.01)^B^). The probability of pelvis/hip contact (0.11 (0.01)^C^) was significantly smaller than the probability of head contact (0.19 (0.01)^B^). Two superscript letters indicate a body part that did not differ from two other body parts that differed between themselves in probability of contact. For example, the probability of shoulder contact (0.08 (0.01)^CD^) was not different than the probability of pelvis/hip contact (0.11 (0.01)^C^), indicated by the same letter C, and also was not different than the probability of contact to the knee (0.06 (0.01)^D^), indicated by the same letter D. However, there was a significant difference in the probability of contact to the knee (0.06 (0.01)^D^) and pelvis/hip (0.11 (0.01)^C^)

The probability of contact to objects depended on fall direction, and fall direction interacted with the probability of body part contacts to objects (*p < *0.001; Table 3). Intentional hand/wrist contact with objects was more common during forward and sideways than during backward falls (*p ≤ *0.014). The head was less likely to impact an object during forward than backward (*p =* 0.001) or sideways falls (*p =* 0.003). Impact of the torso with objects was more common during backward than sideways or forward falls (*p < *0.001).

The probability of contact to objects depended on age (Table 3). Participants older than the median age were more likely than participants younger than the median age to experience contacts to the head (*p =* 0.012), torso (*p =* 0.001), and elbow/forearm (*p =* 0.004). There was also a higher probability of head contacts to objects in females than males (0.18 ± 0.01 versus 0.15 ± 0.01, *p =* 0.028).

### Types of contacted objects

**Fig. 5:**
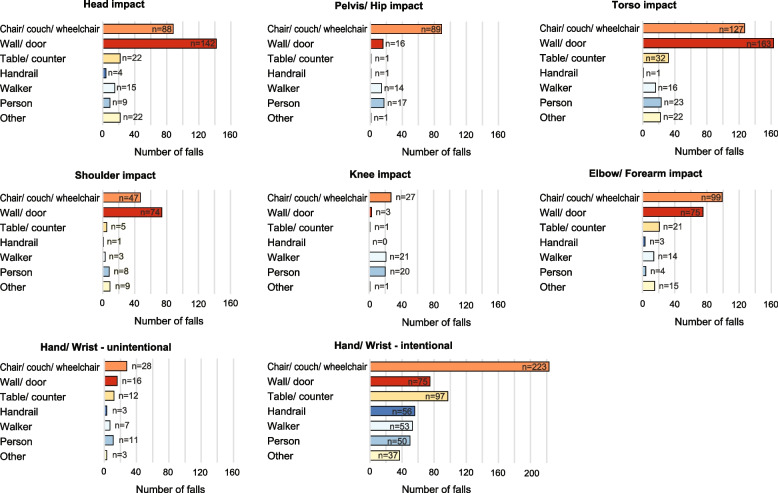
Distribution of impacted objects by body parts during falls in the event of impact

The types of objects most often contacted depended on the body part (Fig. 5). The most common object contacted by the head, torso and shoulder was a wall. The most common object contacted by the hand/wrist (both intentionally and unintentionally), elbow/forearm and pelvis/hip was a chair/couch/wheelchair. The next most common objects to be contacted intentionally by the hand/wrist were table/counter, wall/door, handrail, walker/rollator and another person.

## Discussion

Safe environments are essential for promoting mobility and preventing falls and fall-related injuries in older adults. A barrier to the design of safe movement environments is lack of evidence on how environmental features act as hazards or safeguards for falls [5, 6]. In the current study, we provide objective evidence on how older adults interact with objects (and other people) before, during, and after falls, based on analysis of video footage of real-life falls in LTC. We found that most falls in common areas of LTC involved interactions with objects other than the floor. We also found that intentional interactions with objects were more common during forward falls, whereas unintentional impacts with objects were more common during backward falls. These direction-dependant differences suggest that the ability to visualize the falling environment has a role in adjustment of falling patterns to environmental objects.

In line with previous findings [13], participants held objects before the onset of imbalance in over one-half of falls. The most commonly held objects were walkers (19% of falls, Fig. 2d, e) and chairs (17% of falls). Participants held handrails at the onset of only 2% of falls. These trends either reflect the value of handrails in protecting against falls, or the relatively small amount of time that participants held handrails. In support of the former, falls often occurred while crossing hallway intersections, where participants needed to release their grip on a handrail to cross the intersection (e.g., Fig. 2h). However, we cannot determine whether it was safer for participants to hold handrails than walkers or chairs because we had no measures of the amount of time people held various objects.

Interactions with objects or other people had a central role in causing about one third of falls. These interactions most often involved chairs, wheelchairs, or walkers, which participants either tripped upon while walking, or lost contact with while standing or walking. Our results expand previous work [16, 31] by showing how specific objects contributed to falls, and highlight the importance of further research on the design of chairs and walkers that are less likely to cause trips, or suddenly give way and create falls due to loss of support. We also found that 13% of falls were due to being pushed or pulled by another person. This observation supports the need for improved strategies for reducing aggressive behaviour to reduce falls in the LTC setting [32, 33].

In 60% of falls, participants came into contact with objects after the onset of the fall. In 45% of all falls, there were unintentional impacts between body parts and objects other than the floor, most notably to the head, which was the third most common body part to impact objects, after the hands and torso. Indeed, the head was nearly as likely to impact an object, as it was to impact the floor (42% of all head impacts were to objects). The most common object for the head to impact was a wall, followed by a chair or couch. Of falls that involved contacts to objects, contacts to walls were over 2-fold more likely to involve the head, torso, or shoulder than the pelvis or hands. These results reflect that falls tended to occur nearby but not directly adjacent to walls.

Nearly one-third of all falls (31%) involved intentional contacts to objects by the hands, either through reach-to-grasp or bracing after the onset of the fall (falls (a, c) of Fig. 2). These findings show that older adults adjusted their fall-protective responses to features of the environment [34, 35]. Of note, hand contacts to handrails were relatively rare – again perhaps reflecting the value of handrail grasping for maintaining balance and preventing falls. Hand contacts to chairs and couches were nearly 4 times more common than to handrails; hand contacts to tables and counters were twice as common as to handrails (see falls (a), (b) and (i) in Fig. 2).

The speed and accuracy of reach-to-grasp responses for balance recovery depend on both visual mapping before the onset of imbalance, and online visual information after the perturbation [34–36]. Our results provide several lines of evidence supporting the importance of visual mapping on the mechanics of falls. First, intentional hand contacts to objects (reach-to-grasp and bracing) were most common in forward falls (as shown in fall (a) of Fig. 2), where participants were likely better able to visualize and tailor their falling movements to objects in the path of the fall, than they would have been in backward or sideways falls. In contrast, backward and sideways falls were more likely to involve impact of the elbows with nearby objects (as shown in fall (c) of Fig. 2). Second, unintentional impacts by the head and torso to objects were more common in backward and sideways falls than in forward falls (as shown in falls (c) and (d) of Fig. 2), perhaps because participants were less able to visualize and coordinate their falling patterns to nearby environmental features in backward and sideways falls. Interventions to enhance visual attention and mapping strategies [37, 38] may have an important role in both preventing and reducing the severity of falls. At the same time, it is important to recognize the high demands that balance recovery by grasping places on reaction time as well as on upper limb and core muscle strength and flexibility, all of which are often impaired in older adults [39, 40]. Examining these factors was outside of the scope of our study, and further research is warranted to characterize the physiological demands required for interactions with objects during falls.

Interactions with objects during falls were common for a wide range of the LTC population that we studied, and were not strongly predicted by clinical or demographic status, with four notable exceptions. First, when compared to participants younger than the median age, participants older than the median age were between 1.6 and 1.9-fold more likely to hold objects prior to the onset of the fall, and contact objects during the fall. Similarly, participants with stroke were 2-fold more likely to hold and unintentionally impact objects while participants with diabetes were 1.8-fold more likely to intentionally contact objects by the hands. These trends may be explained by higher use of mobility aids in older individuals [41] and in individuals with stroke, or the tendency for individuals who were older or with greater mobility impairment to stay in close proximity to objects (e.g., furniture walking) for perceived safety. Finally, the opposite trends were observed for participants who were taking antipsychotics, who were 1.3-fold less likely to contact objects before and during falls.

Our observation that 60% of falls involved contacts between body parts and objects other than the floor during falls has implications for the design and evaluation of strategies to prevent falls and fall-related injuries. Efforts to prevent falls and fall-related injuries through the removal of environmental hazards in private homes, and installation of protective features such as handrails and anti-slip mats, have yielded mixed results, with some studies showing a reduction in falls [42, 43] and fall-related injuries [44], and others showing no effect [7]. Also, efforts to prevent fall-related injuries in LTC with compliant flooring (designed to cushion the impact of the fall) have yielded mixed results, with a recent randomized controlled trial showing no effect on falls and fall-related injuries [12]. In attempting to explain these results, Mackey et al. noted “it was not possible to know which body parts experienced impact during falls and whether there was impact with walls, furniture, or other objects during fall descent that would render floor stiffness less important in determining risk for injury”. Our results support Mackey et al.’s suggestion that the geometry and stiffness of the furniture and walls may be as important as reducing the stiffness of the floor to prevent fall-related injuries in the LTC environment [12].

Our results also have implications for the design and evaluation of systems for automatically detecting falls based on wearable sensors or video cameras [45–48]. Most research in this field has trained and evaluated fall detection algorithms based on laboratory-based falls in “empty rooms,” which accounted for 19% of falls in our study. Our results highlight the need to develop and evaluate these systems for falls in cluttered environments.

Our study has several limitations. First, we examined environmental interactions with objects in common areas of LTC. The prevalence of nearby objects and the interaction with these objects might be different in other environments, such as in private bedrooms, bathrooms, staircases or outdoors. Second, our results might not generalize to other older adult populations, such as community dwelling older adults or those in assisted living; populations who might have different falling patterns and better cognitive and ADL status. However, we did not find differences in cognitive status between falls with and without contacted objects. Third, we did not explore the location of the objects and their distance relevant to participants, factors that likely affect the odds for sustaining impact with objects. Fourth, unlike initially forward, backward, and sideways falls, we classified straight-down falls based on the body configuration at landing. We repeated the analysis with straight-down falls excluded, or considered as a separate category. When compared to the results we have reported, we found no differences in our study conclusions. However, intentional hand contacts with objects were no longer more common in sideways than backward falls, and intentional hand contacts with objects became more frequent in forward than sideways falls. Furthermore, while we classified hand contacts to objects as intentional or unintentional, we regarded all contacts to objects by body parts other than the hands as unintentional. While it is unlikely that body parts such as the head, torso or pelvis intentionally impacted objects, it is possible that some of the elbow or forearm contacts with objects were intentional, and this merits further investigation. Additionally, we did not examine the forces generated during body-part contacts with objects. Future studies might examine the feasibility of measuring contact forces with sensors integrated into objects that are commonly contacted in the LTC setting. Finally, we did not consider the injury consequences of falls. We instead focused on documenting the frequency and nature of human interactions with environmental objects during falls in LTC. This is an important step in the development of safe movement environments for older adults. An important next step is to examine how different types of human-environmental interactions affect the odds for injury during a fall.

## Conclusions

Our study provides objective evidence on how older adults interact with environmental objects and other people during falls, and on the high prevalence of these interactions. Overall, 81% of falls from standing height in LTC involved interactions with objects, reflecting the importance of considering environmental objects in the design and evaluation of strategies to prevent falls and fall-related injuries. Intentional interactions with objects were more common during forward falls, while unintentional impacts with objects were most frequent during backward falls, supporting the importance of visual mapping on the mechanics of falls.

### Supplementary Information

Additional file 1. Odds that a participant would contact objects while falling forward. A table that describes the odds that a participant would fall forward at least once between environments, and differences in the average number of falls between environments.

Additional file 2. Odds that a participant would contact objects while falling backward. A table that describes the odds that a participant would fall backward at least once between environments, and differences in the average number of falls between environments.

Additional file 3. Odds that a participant would contact objects while falling sideways. A table that describes the odds that a participant would fall sideways at least once between environments, and differences in the average number of falls between environments.

## Data Availability

The datasets analyzed during the current study are available from the corresponding authors on reasonable request.

## Abbreviations

ADL: Activities of Daily Living
CPS: Cognitive Performance Scale
GEE: Generalized Estimating Equations
LTC: Long-Term Care
MDS: Minimum Data Set

## References

[1] World Health Organization, Falls. https://www.who.int/news-room/fact-sheets/detail/falls. Accessed 29 Feb 2024.

[2] Harvey LA, Mitchell R, Brodaty H, Draper B, Close JC. Comparison of fall-related traumatic brain injury in residential aged care and community-dwelling older people: a population-based study. Australas J Ageing. 2017;36(2):144–50. 28635089 10.1111/ajag.12422

[3] Rubenstein LZ, Josephson KR, Osterweil D. Falls and fall prevention in the nursing home. Clin Geriatr Med. 1996;12(4):881–902. 8890121 10.1016/S0749-0690(18)30206-4

[4] Australian Institute of Health & Welfare. Trends in hospitalised injury due to falls in older people 2007–08 to 2016–17. In: Government As, editor. Injury Research and Statistics Series. 2019.

[5] Carter SE, Campbell EM, Sanson-Fisher RW, Redman S, Gillespie WJ. Environmental hazards in the homes of older people. Age Ageing. 1997;26(3):195–202. 9223715 10.1093/ageing/26.3.195

[6] Jiang Y, Xia Q, Zhou P, Jiang S, Diwan VK, Xu B. Environmental hazards increase the fall risk among residents of long-term care facilities: a prospective study in Shanghai, China. Age Ageing. 2021;50(3):875–81. 33150929 10.1093/ageing/afaa218

[7] Lord SR, Menz HB, Sherrington C. Home environment risk factors for falls in older people and the efficacy of home modifications. Age Ageing. 2006;35(Suppl 2):ii55–ii19. 16926207 10.1093/ageing/afl088

[8] Tanaka T, Matsumoto H, Son BK, Imaeda S, Uchiyama E, Taniguchi S, et al. Environmental and physical factors predisposing middle-aged and older Japanese adults to falls and fall-related fractures in the home. Geriatr Gerontol Int. 2018;18(9):1372–7. 30133136 10.1111/ggi.13494

[9] Connell BR, Wolf SL. Environmental and behavioral circumstances associated with falls at home among healthy elderly individuals. Atlanta FICSIT Group. Arch Phys Med Rehabil. 1997;78(2):179–86. 9041900 10.1016/S0003-9993(97)90261-6

[10] Yang Y, Feldman F, Leung PM, Scott V, Robinovitch SN. Agreement between video footage and fall incident reports on the circumstances of falls in long-term care. J Am Med Dir Assoc. 2015;16(5):388–94. 25669670 10.1016/j.jamda.2014.12.003

[11] Komisar V, Maki BE, Novak AC. Effect of handrail height and age on the timing and speed of reach-to-grasp balance reactions during slope descent. Appl Ergon. 2019;81:102873. 31422250 10.1016/j.apergo.2019.102873

[12] Mackey DC, Lachance CC, Wang PT, Feldman F, Laing AC, Leung PM, et al. The Flooring for Injury Prevention (FLIP) study of compliant flooring for the prevention of fall-related injuries in long-term care: a randomized trial. PLoS Med. 2019;16(6):e1002843. 31233541 10.1371/journal.pmed.1002843 PMC6590787

[13] Komisar V, Shishov N, Yang Y, Robinovitch SN. Effect of holding objects on the occurrence of head impact in falls by older adults: evidence from real-life falls in long-term care. J Gerontol A Biol Sci Med Sci. 2021;76(8):1463–70. 32622345 10.1093/gerona/glaa168 PMC8277085

[14] Komisar V, Dojnov A, Yang Y, Shishov N, Chong H, Yu Y, et al. Injuries from falls by older adults in long-term care captured on video: prevalence of impacts and injuries to body parts. BMC Geriatr. 2022;22(1):343. 35439948 10.1186/s12877-022-03041-3 PMC9019961

[15] Santello M. Review of motor control mechanisms underlying impact absorption from falls. Gait Posture. 2005;21(1):85–94. 15536038 10.1016/j.gaitpost.2004.01.005

[16] Robinovitch SN, Feldman F, Yang Y, Schonnop R, Leung PM, Sarraf T, et al. Video capture of the circumstances of falls in elderly people residing in long-term care: an observational study. Lancet (London, England). 2013;381(9860):47–54. 23083889 10.1016/S0140-6736(12)61263-X PMC3540102

[17] Yang Y, Schonnop R, Feldman F, Robinovitch SN. Development and validation of a questionnaire for analyzing real-life falls in long-term care captured on video. BMC Geriatr. 2013;13:40. 23635343 10.1186/1471-2318-13-40 PMC3655003

[18] Yang Y, Komisar V, Shishov N, Lo B, Korall AM, Feldman F, et al. The effect of fall biomechanics on risk for hip fracture in older adults: a cohort study of video-captured falls in long-term care. J Bone Miner Res. 2020;35(10):1914–22. 32402136 10.1002/jbmr.4048 PMC7689902

[19] Norton R, Campbell AJ, Lee-Joe T, Robinson E, Butler M. Circumstances of falls resulting in hip fractures among older people. J Am Geriatr Soc. 1997;45(9):1108–12. 9288020 10.1111/j.1532-5415.1997.tb05975.x

[20] Parkkari J, Kannus P, Palvanen M, Natri A, Vainio J, Aho H, et al. Majority of hip fractures occur as a result of a fall and impact on the greater trochanter of the femur: a prospective controlled hip fracture study with 206 consecutive patients. Calcif Tissue Int. 1999;65(3):183–7. 10441647 10.1007/s002239900679

[21] Fleiss JL. Statistical methods for rates and proportions. New York: John Wiley and Sons; 1981.

[22] Hawes C, Morris JN, Phillips CD, Fries BE, Murphy K, Mor V. Development of the nursing home Resident Assessment Instrument in the USA. Age Ageing. 1997;26(Suppl 2):19–25. 9464550 10.1093/ageing/26.suppl_2.19

[23] Morris JN, Fries BE, Morris SA. Scaling ADLs within the MDS. J Gerontol A Biol Sci Med Sci. 1999;54(11):M546–53. 10619316 10.1093/gerona/54.11.M546

[24] Morris JN, Fries BE, Mehr DR, Hawes C, Phillips C, Mor V, et al. MDS cognitive performance scale. J Gerontol. 1994;49(4):M174–82. 8014392 10.1093/geronj/49.4.M174

[25] Robinovitch SN, Dojnov A, Komisar V, Yang Y, Shishov N, Yu Y, et al. Protective responses of older adults for avoiding injury during falls: evidence from video capture of real-life falls in long-term care. Age Ageing. 2022;51(12):afac273. 36477785 10.1093/ageing/afac273 PMC9729006

[26] Te B, Komisar V, Aguiar OMG, Shishov N, Robinovitch SN. Compensatory stepping responses during real-life falls in older adults. Gait Posture. 2023;100:276–83. 36689855 10.1016/j.gaitpost.2023.01.005

[27] Komisar V, van Schooten KS, Aguiar OMG, Shishov N, Robinovitch SN. Circumstances of falls during sit-to-stand transfers in older people: a cohort study of video-captured falls in long-term care. Arch Phys Med Rehabil. 2023;104(4):533–40. 36402204 10.1016/j.apmr.2022.10.012

[28] Gryfe CI, Amies A, Ashley MJ. A longitudinal study of falls in an elderly population: I. Incidence and morbidity. Age Ageing. 1977;6(4):201–10. 596307 10.1093/ageing/6.4.201

[29] Stevens JA, Sogolow ED. Gender differences for non-fatal unintentional fall related injuries among older adults. Inj Prev. 2005;11(2):115–9. 15805442 10.1136/ip.2004.005835 PMC1730193

[30] Sadigh S, Reimers A, Andersson R, Laflamme L. Falls and fall-related injuries among the elderly: a survey of residential-care facilities in a Swedish municipality. J Community Health. 2004;29(2):129–40. 15065732 10.1023/B:JOHE.0000016717.22032.03

[31] Holliday PJ, Fernie GR, Gryfe CI, Griggs GT. Video recording of spontaneous falls of the elderly. In: Gray BE, Ed. Slips, Stumbles, and Falls: Pedestrian Footwear and Surfaces (ASTM STP 1103). Philadelphia: American Society for Testing and Materials; 1990. p. 7–16.

[32] Teresi JA, Ramírez M, Fulmer T, Ellis J, Silver S, Kong J, et al. Resident-to-resident mistreatment: evaluation of a staff training program in the reduction of falls and injuries. J Gerontol Nurs. 2018;44(6):15–23. 29677382 10.3928/00989134-20180326-01 PMC6668910

[33] Murphy B, Bugeja L, Pilgrim J, Ibrahim JE. Deaths from resident-to-resident aggression in Australian nursing homes. J Am Geriatr Soc. 2017;65(12):2603–9. 29131309 10.1111/jgs.15051

[34] Cheng KC, McKay SM, King EC, Maki BE. Does aging impair the capacity to use stored visuospatial information or online visual control to guide reach-to-grasp reactions evoked by unpredictable balance perturbation? J Gerontol A Biol Sci Med Sci. 2012;67(11):1238–45. 22511290 10.1093/gerona/gls116

[35] King EC, McKay SM, Lee TA, Scovil CY, Peters AL, Maki BE. Gaze behavior of older adults in responding to unexpected loss of balance while walking in an unfamiliar environment: a pilot study. J Optom. 2009;2(3):119–26. 10.3921/joptom.2009.119

[36] Ghafouri M, McIlroy WE, Maki BE. Initiation of rapid reach-and-grasp balance reactions: is a pre-formed visuospatial map used in controlling the initial arm trajectory? Exp Brain Res. 2004;155(4):532–6. 14985902 10.1007/s00221-004-1855-8

[37] Reed-Jones RJ, Dorgo S, Hitchings MK, Bader JO. Vision and agility training in community dwelling older adults: incorporating visual training into programs for fall prevention. Gait Posture. 2012;35(4):585–9. 22206782 10.1016/j.gaitpost.2011.11.029 PMC3405148

[38] Koppelaar H, Kordestani-Moghadam P, Kouhkani S, Irandoust F, Segers G, de Haas L, et al. Proof of concept of novel visuo-spatial-motor fall prevention training for old people. Geriatrics (Basel). 2021;6(3):66. 34210015 10.3390/geriatrics6030066 PMC8293049

[39] Adams K, O’Shea P, O’Shea KL. Aging: its effects on strength, power, flexibility, and bone density. Strength Cond J. 1999;21(2):65.

[40] Mattay VS, Fera F, Tessitore A, Hariri AR, Das S, Callicott JH, et al. Neurophysiological correlates of age-related changes in human motor function. Neurology. 2002;58(4):630–5. 11865144 10.1212/WNL.58.4.630

[41] Gell NM, Wallace RB, LaCroix AZ, Mroz TM, Patel KV. Mobility device use in older adults and incidence of falls and worry about falling: findings from the 2011–2012 national health and aging trends study. J Am Geriatr Soc. 2015;63(5):853–9. 25953070 10.1111/jgs.13393 PMC4439269

[42] Gillespie LD, Robertson MC, Gillespie WJ, Sherrington C, Gates S, Clemson LM, et al. Interventions for preventing falls in older people living in the community. Cochrane Database Syst Rev. 2012;2012(9):Cd007146. 22972103 10.1002/14651858.CD007146.pub3 PMC8095069

[43] Campbell AJ, Robertson MC, La Grow SJ, Kerse NM, Sanderson GF, Jacobs RJ, et al. Randomised controlled trial of prevention of falls in people aged > or =75 with severe visual impairment: the VIP trial. BMJ. 2005;331(7520):817. 16183652 10.1136/bmj.38601.447731.55 PMC1246082

[44] Keall MD, Pierse N, Howden-Chapman P, Cunningham C, Cunningham M, Guria J, et al. Home modifications to reduce injuries from falls in the home injury prevention intervention (HIPI) study: a cluster-randomised controlled trial. Lancet (London, England). 2015;385(9964):231–8. 25255696 10.1016/S0140-6736(14)61006-0

[45] Martínez-Villaseñor L, Ponce H, Brieva J, Moya-Albor E, Núñez-Martínez J, Peñafort-Asturiano C. UP-fall detection dataset: a multimodal approach. Sensors (Basel). 2019;19(9):1988. 31035377 10.3390/s19091988 PMC6539235

[46] Cotechini V, Belli A, Palma L, Morettini M, Burattini L, Pierleoni P. A dataset for the development and optimization of fall detection algorithms based on wearable sensors. Data Brief. 2019;23:103839. 31372467 10.1016/j.dib.2019.103839 PMC6660610

[47] Casilari E, Lora-Rivera R, García-Lagos F. A study on the application of convolutional neural networks to fall detection evaluated with multiple public datasets. Sensors (Basel). 2020;20(5):1466. 32155936 10.3390/s20051466 PMC7085732

[48] Aziz O, Musngi M, Park EJ, Mori G, Robinovitch SN. A comparison of accuracy of fall detection algorithms (threshold-based vs. machine learning) using waist-mounted tri-axial accelerometer signals from a comprehensive set of falls and non-fall trials. Med Biol Eng Comput. 2017;55(1):45–55. 27106749 10.1007/s11517-016-1504-y

