# Supplementary material for: Interactions during falls with environmental objects: evidence from real-life falls in long-term care captured on video
Source: BMC Geriatr. 2024 Sep 2;24:726. doi: 10.1186/s12877-024-05306-5 (PMC11368007; doi:10.1186/s12877-024-05306-5)
Supplement: Supplementary file 1 — Additional file 1. Odds that a participant would contact objects while falling forward. A table that describes the odds that a participant would fall forward at least once between environments, and differences in the average number of falls between environments. [file 12877_2024_5306_MOESM1_ESM.docx]

**Additional file 1.** Odds that a participant would contact objects while falling forward.

| Environment | Number of falls | Frequency (% of falls) | Proportion (of the 236 participants) who fell forward at least once | Odds ratio (95%CI) | P value | Average number of falls per participant | Ratio of counts  (95%CI) | P value |
| --- | --- | --- | --- | --- | --- | --- | --- | --- |
| **(a)** |  |  |  |  |  |  |  |  |
| Contacted objects after fall initiation ^j, k^ | 213 | 50.7 | 0.61 (0.54-0.67) | 0.91 (0.63-1.33) | p=0.636 | 0.90 (0.74-1.08) | 1.03 (0.86-1.24) | p=0.761 |
| Did not contact objects after fall initiation ^h^ | 207 | 49.3 | 0.63 (0.56-0.69) | 1 | … | 0.87 (0.74-1.02) | 1 | … |
| **(b)** |  |  |  |  |  |  |  |  |
| Did not contact objects after fall initiation ^h^ | 207 | 49.3 | 0.63 (0.56-0.69)^B^ | 5.29 (3.55-7.88) | **p<0.001** | 0.87 (0.74-1.02)^B^ | 3.29 (2.54-4.26) | **p<0.001** |
| Intentionally contacted objects after fall initiation ^j^ | 84 | 20.0 | 0.28 (0.22-0.34)^A^ | 1.19 (0.79-1.81) | p=0.400 | 0.35 (0.27-0.47)^A^ | 1.33 (0.96-1.85) | p=0.085 |
| Intentionally ^j^ AND unintentionally ^k^ contacted objects after fall initiation | 66 | 15.7 | 0.24 (0.19-0.30)^A^ | 0.98 (0.64-1.49) | p=0.914 | 0.28 (0.21-0.36)^A^ | 1.05 (0.76-1.45) | p=0.779 |
| Unintentionally impacted  objects after fall initiation ^k^ | 63 | 15.0 | 0.24 (0.19-0.30)^A^ | 1 | … | 0.26 (0.21-0.34)^A^ | 1 | … |
| **(c)** |  |  |  |  |  |  |  |  |
| Contacted objects after fall initiation ^j, k^ AND held  object ^i^ | 90 | 21.4 | 0.31 (0.26-0.38)^AB^ | 0.73 (0.50-1.06) | p=0.101 | 0.38 (0.31-0.47)^A^ | 0.73 (0.55-0.97) | **p=0.031** |
| Contacted objects after fall initiation ^j, k^ AND did not hold  object ^g^ | 123 | 29.3 | 0.35 (0.29-0.41)^AB^ | 0.86 (0.59-1.26) | p=0.445 | 0.52 (0.40-0.67)^B^ | 1.00 (0.77-1.29) | p=0.997 |
| Did not contact objects after fall initiation ^h^ AND held object ^i^ | 84 | 20.0 | 0.29 (0.23-0.35)^A^ | 0.65 (0.44-0.95) | **p=0.025** | 0.35 (0.28-0.45)^A^ | 0.68 (0.50-0.94) | **p=0.018** |
| Did not contact objects after fall initiation ^h^ AND did not hold object ^g^ | 123 | 29.3 | 0.39 (0.33-0.45)^B^ | 1 | … | 0.52 (0.42-0.64)^B^ | 1 | … |

Notes:

-Significant differences (p < 0.05) in the odds that a participant would fall at least once between environments, and differences in the average number of falls between environments are **bolded**.

-Superscripts capital letters indicate the results of statistical comparisons between environmental classifications for the column of interest. Environmental classifications that differed significantly (p < 0.05) in contact probability to any surface are indicated by different letters; environmental classifications that did not differ significantly (p > 0.05) in contact probability to any surface are indicated by the same letter. The sequence of the superscript letters is from lowest to highest proportions and average number of falls. For example, the proportion of participants who fell at least once and “Did not contact objects after fall initiation AND held object” (0.29 (0.23-0.35)^A^) was significantly smaller than the proportion of participants who fell at least once and “Did not contact objects after fall initiation AND did not hold object” (0.39 (0.33-0.45)^B^). Two superscript letters indicate an environment that did not differ from two other environments in the proportion of participants who fell at least once. For example, the proportion of participants who fell at least once and “Contacted objects after fall initiation AND did not hold object” (0.35 (0.29-0.41)^AB^) was not different than the proportion of participants who fell at least once and “Did not contact objects after fall initiation AND held object" (0.29 (0.23-0.35)^A^), indicated by the same letter A, and also was not different than the proportion of participants who fell at least once and “Did not contact objects after fall initiation AND did not hold object” (0.39 (0.33-0.45)^B^), indicated by the same letter B. However there was a significant difference in the proportion of participants who fell at least once and “Did not contact objects after fall initiation AND held object” (0.29 (0.23-0.35)^A^) and “Did not contact objects after fall initiation AND did not hold object” (0.39 (0.33-0.45)^B^).

-Superscript lower case letters indicate the types of interactions with objects included in each category:

^g^ falls that did not involve held objects at the time of fall initiation

^h^ falls that did not involve any interactions or contacts to objects after fall initiation (may have involved held objects at the time of fall initiation)

^i^ falls that involved held objects at the time of fall initiation

^j^ falls that involved hand contacts to objects after fall initiation that appeared to be intentional, including reach-to-grasp movements or bracing of the hands on objects to arrest the fall (in comparisons (a) and (b), these falls may have also involved held objects at the time of fall initiation)

^k^ falls that involved impact after fall initiation between objects and any part of the body (e.g. head, torso, shoulder, pelvis/hip, knee, elbow/forearm, and hand/wrist), that were not due to reach-to-grasp movements or hand bracing, and generally appeared to be unintentional (in comparisons (a) and (b), these falls may also have involved held objects at the time of fall initiation)
